# Activism and scientific research: 20 years of community action by the Vancouver area network of drug users

**DOI:** 10.1186/s13011-018-0158-1

**Published:** 2018-05-22

**Authors:** Ehsan Jozaghi, Alissa M. Greer, Hugh Lampkin, Jane A. Buxton

**Affiliations:** 1 The British Columbia Centre for Disease Control, 655 W 12th Ave, Vancouver, BC V5Z 4R4 Canada; 2 The School of Population and Public Health, Faculty of Medicine, University of British Columbia, 2206 East Mall, Vancouver, BC V6T 1Z3 Canada; 3 Vancouver Area Network of Drug Users, 380 E Hastings St, Vancouver, BC V6A 1P4 Canada

## Abstract

**Background:**

Over the past several decades, there have been numerous peer-reviewed articles written about people who use drugs (PWUDs) from the Downtown Eastside neighborhood of Vancouver, Canada. While individual researchers have engaged and acknowledged this population as participants and community partners in their work, there has been comparatively little attention given to the role of PWUDs and drug user organizations in directing, influencing, and shaping research agendas.

**Methods:**

In this community-driven research, we examine 20 years of peer-reviewed studies, university theses, books, and reports that have been directed, influenced, and shaped by members of the activist organization the Vancouver Area Network of Drug Users (VANDU). In this paper, we have summarized VANDU’s work based on different themes from each article.

**Results:**

After applying the inclusion criteria to over 400 articles, 59 items containing peer-reviewed studies, books, and reports were included and three themes of topics researched or discussed were identified. Theme 1: ‘health needs’ of marginalized groups was found in 39% of articles, Theme 2: ‘evaluation of projects’ related to harm reduction in 19%, and Theme 3: ‘activism’ related work in 42%. Ninety-four percent of co-authors were from British Columbia and 44% of research was qualitative. Works that have been co-authored by VANDU’s members or acknowledged their participations created 628 citations. Moreover, their work has been accessed more than 149,600 times.

**Conclusions:**

Peer-based, democratic harm reduction organizations are important partners in facilitating groundbreaking health and social research, and through research can advocate for the improved health and wellbeing of PWUDs and other marginalized groups in their community. This article also recommends that PWUDs should be more respectfully engaged and given appropriate credit for their contributions.

## Background

More than 20 years ago, the city of Vancouver, Canada, was experiencing severe overdose and blood-borne disease epidemics concentrated in the Downtown Eastside (DTES) neighbourhood [1, 2]. These epidemics were attributed to the ineffective and inefficient health and social policies tailored toward the people who use drugs (PWUDs) [3, 4]. The Vancouver Area Network of Drug Users (VANDU) was formed to address the gross injustice of health and human rights toward PWUDs [5]. Once considered a fringe organization by many, the membership of VANDU has grown substantially and VANDU is now fully funded through the local health authority, Vancouver Coastal Health [6]. For the range of work they have done in averting blood borne infections, overdose death prevention, and other pioneering harm reduction projects, VANDU has been the recipient of a number of awards by various non-government and government organizations [7, 8].

Since its formation, VANDU has played a critical role in research by virtue of its familiarity with the issues that affect the health of PWUDs and the organization’s ability to facilitate connections between researchers and PWUD. Many of VANDU’s members reside in the DTES and are affected by poverty, social marginalization, homelessness, substance use disorders, mental health issues, and structural violence [9–14]. Over the past few decades, there have been numerous peer-reviewed studies and reports conducted on the marginalized and at risk population in the DTES. However, scant attention has been given to the collective role of PWUDs and the power of advocacy by marginalized members of society in influencing their health and well-being.

In 2017, a VANDU board member approached the first author for assistance to describe studies with which VANDU members and the organization had been involved in to feature the publications on VANDU’s website for their twentieth anniversary. Therefore, we identified peer-reviewed studies, university theses, books, book chapters, and reports that have been directed, influenced, and shaped by VANDU members. This research characterizes these articles by theme, methodology, and social media impact in the scientific and greater community.

## Methods

The primary objective and scope of this study was to compile and summarize a review of the research and policy projects that VANDU has been involved and acknowledged in. The key indicators of interest were authorship, publication type, method, impact, and subject matter, or ‘theme,’ of the publication. In order to identify relevant publications and information, we searched Google Scholar since it is known to capture not only all peer-reviewed studies that Medline, Web of Science, Scopus or Pubmed often miss, but also theses, reports, and other gray literature. In addition, Google Scholar better captures the citation impact of articles since Medline, Pubmed, Web of Science, and Scopus are not focused on reports, theses, books, and book chapters. For this article, we focus on projects and papers that VANDU had been involved in and published up until February 26, 2018.

The Google Scholar search column, “Vancouver Area Network of Drug Users” was searched with no restrictions on the publication year from the first page of search engine results to the last page because the search engine “reports with no details of the means by which … [the results] are ordered” [15]. Publications were included if they either identified “Vancouver Area Network of Drug Users” or a VANDU member as a co-author, or thanked the membership for their direct contribution to the project. As well, publications were only included if they were written in the English language.

The studies that met the initial assessment of VANDU authorship or direct involvement were reviewed in full. Study content was summarized, and methodology, impact, author’s affiliation, and role (researcher, doctoral student or VANDU/community partner) at the time of the research were identified. The geographic regions in which the co-authors were based at the time of the research/publication were also identified.

Extracted data was compiled into tables and the study content was explored to identify topic themes. The themes were derived by reviewing each study and identifying commonalities between articles and their overall objectives. The themes identified are mutually exclusive because each theme is unique, representing work that VANDU as an author organization or a supporting partner has been able to achieve through their research activities.

Study methodology was categorized as qualitative, quantitative, or other. Several measures of media impact were generated from the journals, universities, and sites that report downloads and social media impacts such as Altmetric scores (for BMC, Taylor & Francis, Wiley, Springer and SAGE journals) and PlumX metrics (for Elsevier journals). Altmetric and PlumX metrics are used to measure the broader research impacts in social web via different non-academic sources, such as news articles citing the study, blogs, Wikipedia, video uploads, Twitter posts, Mendeley users, Google+ posts, Reddit, and Facebook shares [16–18]. Citation counts of the studies were captured via Google Scholar. The information related to articles (e.g., research, opinion, and letter), and thesis downloads/views was accessed from the journal or university websites.

A printed summary of identified articles were presented to the VANDU board in August, 2017; the board decided to pursue an academic publication, discussed the concept for this manuscript, and identified HL as VANDU’s appointed author. A printed draft manuscript was presented to VANDU for input in November, 2017; subsequently VANDU members, including author HL, presented preliminary results at three local conferences and lecture series. Members of the board were supported in these knowledge translation activities by receiving poster printing costs, transportation, registration fees and honoraria. EJ facilitated discussions with VANDU board members as they reviewed printed updated manuscript drafts to provide further feedback which was incorporated prior to article submission and in the response to reviewers. In January 2018, VANDU’s appointed author (HL) and the board members were provided with an updated printed summary of the identified articles. In addition, an updated printed draft of the manuscript and tables were given to VANDU board members for further feedback in March 2018.

## Results

The literature search identified more than 400 articles. A total of 341 articles were excluded because they were determined from the review of title, abstract and body not to be relevant. A total of 59 articles containing peer-reviewed studies, books, book chapters, theses, and reports were identified. Three themes of topics researched or discussed in the articles were identified. Theme 1, ‘health needs,’ was based on the health and wellbeing of marginalized groups (e.g. sex workers, PWUD, illicit drinkers, and DTES residents). Theme 2, ‘evaluation of projects,’ described and evaluated harm reduction projects (e.g., unsanctioned needle distribution, injection rooms, smoking rooms, injection support teams and harm reduction training/education). Finally, Theme 3, ‘activism,’ contained studies that investigated or described the impacts of drug user activism and organizing in the DTES community and beyond. The articles are categorized by author, year of publication, title, type of publication, methodology, and theme in Table 1. Moreover, for research items associated with an academic journal, the impact factor is reported.

**Table 1:** The summary of peer-reviewed studies, reports, theses, books, & book chapters involving VANDU members

Authors	Year	Publication	Type	Method	Theme
Osborn, Boyd & Columbia [44]	1998	Environment and Planning: Society and Space (2.031)^*₸^	Research	Other	3
Osborn [45]	1999	Arsenal Pulp Press ^ǂ^	Book	Other	3
Kerr et al. [46]	2001	Health Canada	Report	Qualitative	3
Rossi & Pacey [47]	2002	Canadian HIV/AIDS Policy & Law Review	Health News	Other	2
Wood et al. [48]	2003	Journal of Urban Health (1.959)	Research	Quantitative	2
Kerr et al. [49]	2003	Journal of Drug Issues (1.161)	Research	Quantitative	3
Silversides [50]	2004	Canadian Medical Association Journal (6.784)	Research	Other	3
Alleyne et al. [51]	2004	Health Canada and the Canadian Centre on Substance Abuse	Report	Other	3
Jürgens [52]	2005	International HIV/AIDS Alliance & Canadian HIV/AIDS Legal Network	Report	Other	3
Kerr et al. [53]	2005	Journal of Urban Health (1.959)	Research	Quantitative	2
Kerr et al. [8]	2006	International Journal of Drug Policy (3.479)	Research	Qualitative	3
Shannon et al. [54]	2006	Harm Reduction Journal (1.880)	Research	Quantitative	1
Osborn & Small [55]	2006	International Journal of Drug Policy (3.479)	Research	Other	1
De Sousa [56]	2006	University of British Columbia: PhD Dissertation	Thesis	Qualitative	1
Banga [57]	2007	University of British Columbia: PhD Dissertation	Thesis	Qualitative	1
Shannon et al. [58]	2008	Substance use & Misuse (1.234)	Research	Quantitative	1
Howard [59]	2008	Simon Fraser University: PhD Dissertation	Thesis	Qualitative	3
Salmon et al. [60]	2009	Women’s Health Research Institute	Report	Qualitative	1
Boyd, MacPherson & Osborn [61]	2009	Fernwood Publication	Book	Other	3
Campbell, Boyd & Culbert [62]	2009	Greystone Books	Book	Other	3
Wilson [63]	2009	Simon Fraser University: PhD Dissertation	Thesis	Qualitative	3
Rachlis et al. [64]	2010	Substance use & Misuse (1.234)	Research	Quantitative	1
Hayashi et al. [25]	2010	International Journal of Drug Policy (3.479)	Research	Quantitative	2
Vancouver Area Network of Drug Users [65]	2010	City of Vancouver	Research	Quantitative	1
Lloyd-Smith [66]	2010	Harm Reduction Journal (1.880)	Research	Quantitative	1
Kruk & Banga [67]	2011	Canadian Journal of Community Mental Health^α^	Research	Qualitative	1
Kendall [68]	2011	Government of British Columbia	Report	Qualitative	1
Reid [69]	2011	Simon Fraser University: Master of Public Policy	Thesis	Qualitative	1
Crabtree et al. [70]	2011	UBC Medical Journal^α^	Research	Qualitative	1
Small et al. [71]	2012	Substance use & Misuse (1.234)	Research	Qualitative	2
McNeil [72]	2013	University of British Columbia: PhD Dissertation	Thesis	Qualitative	1
Grant et al. [73]	2013	UBC Medical Journal^α^	Research	Qualitative	1
Boyd & NAOMI Patients Association [33]	2013	Harm Reduction Journal (1.880)	Research	Qualitative	3
Callon et al. [74]	2013	Harm Reduction Journal (1.880)	Research	Qualitative	2
Ormond [75]	2013	Radical Criminology^α^	Opinion	Other	1
McNeil et al. [27]	2014	AIDS and Behavior (2.916)	Research	Qualitative	2
McNeil et al. [76]	2014	International Journal of Drug Policy (3.479)	Research	Qualitative	1
Jozaghi & Vancouver Area Network of Drug Users [6]	2014	Harm Reduction Journal (1.880)	Research	Other	2
McNeil et al. [77]	2015	International Journal of Drug Policy (3.479)	Research	Qualitative	2
Jozaghi & Vancouver Area Network of Drug Users [28]	2015	Health & Justice^α^	Research	Other	2
Crabtree [78]	2015	University of British Columbia: PhD Dissertation	Thesis	Qualitative	3
Jozaghi [19]	2015	Simon Fraser University: PhD Dissertation	Thesis	Qualitative	3
Himsworth et al. [79]	2015	Vector-Borne and Zoonotic Diseases (2.045)	Research	Quantitative	1
Westfall [80]	2015	Simon Fraser University: Master of Public Policy	Thesis	Qualitative	1
Jozaghi, Lampkin & Andresen [20]	2016	Harm Reduction Journal (1.880)	Research	Qualitative	2
Crabtree et al. [81]	2016	Harm Reduction Journal (1.880)	Research	Qualitative	1
Smith [82]	2016	Intersectionalities: A Global Journal of Social Work Analysis, Research, Polity, and Practice	Research	Other	3
Greer et al. [83]	2016	British Columbia Centre for Disease Control	Report	Other	3
Goodman et al. [84]	2017	Social Science & Medicine (2.797)	Research	Qualitative	1
Boyd, Murray, SNAP & MacPherson [34]	2017	Harm Reduction Journal (1.880)	Research	Qualitative	3
Jozaghi & Marsh [41]	2017	Canadian Journal of Public Health	Letter	Other	3
Thomson et al. [85]	2017	Addiction	Letter	Other	3
Boyd, Murray & NAOMI Patients Association [86]	2017	Critical Inquiries for Social Justice in Mental Health	Book chapter	Other	3
Damon et al. [36]	2017	Social Science & Medicine (2.797)	Research	Other	3
Greer et al. [87]	2017	British Columbia Centre for Disease Control	Report	Other	3
Bouchard et al. [88]	2018	International Journal of Drug Policy (3.479)	Research	Other	3
Lee et al. [89]	2018	Emerging Infectious Diseases (8.222)	Research	Quantitative	1
Rothenburger et al. [90]	2018	EcoHealth (2.252)	Research	Quantitative	1
Jozaghi et al. [91]	2018	Canadian Journal of Public Health	Letter	Other	3

*The journal impact factors of the publication are represented in the parentheses

^₸^The impact factor of the journal is for the year 2017 and not for the year in which the article was published

^ǂ^The impact factor of the journal is not reported if it is a letter, report, theses, book or book chapter

^α^The noted journals have no reported impact factors

As shown on Tables 1, 39% (*n* = 23) of publications were categorized into Theme 1 (health needs), 19% (*n* = 11) into Theme 2 (evaluation of projects), and 42% (*n* = 25) into Theme 3 (activism). Peer-reviewed research articles represented 56% of the publications (*n* = 33), books or book chapters comprised of 7% (*n* = 4), theses 15% (*n* = 9), and other publications (e.g., Health News, Letters to the editor, Reports, and Opinions) comprised of 22% (*n* = 13). The methodology of the studies, citations, social media impact, and downloads are shown in Table 2 based on their thematic categorizations. Table 2 shows the largest proportion of the articles (44%) were qualitative. Theme 1 had the highest qualitative and quantitative categories, while Theme 3 had the highest ‘other’ methodology. Theme 2 had the lowest number of articles but the highest citations and Altmetric scores. Finally, Themes 2 and 3 had the most accessed articles both greater than 64,000.

**Table 2:** Summarizing the research themes in terms of methodology and the research impact

Research Theme	Qualitative	Quantitative	Others	Citations	Altmetric score	Accessed
Theme 1^*^	12 (52%)	8 (35%)	3 (13%)	161	201	21,341
Theme 2^¶^	5 (46%)	3 (27%)	3 (27%)	248	353	64,004
Theme 3^ǂ^	9 (36%)	1 (4%)	15 (60%)	217	268	64,255
Total	26 (44%)	12 (20%)	21 (36%)	628	822	149,600

*Theme 1 = Health needs of marginalized groups

^¶^Theme 2 = Evaluation of projects

^ǂ^Theme 3 = Impact of drug user activism

Table 3 displays the role and geographic regions of the co-authors. The vast majority of co-authors are from British Columbia where VANDU is located.

**Table 3:** Summarizing the research themes in terms of the geographic affiliations and coauthors’ roles

Research Theme	B.C. co-authors	Rest of Canada co-authors	International co-authors	Senior, Junior, & other Researchers	VANDU & the community partners^₸^	Student/Postdoctoral
Theme 1^*^	106 (90%)	9 (7%)	3 (3%)	50 (42%)	39 (33%)	29 (25%)
Theme 2^¶^	41 (100%)	0 (0%)	0 (0%)	20 (49%)	12 (29%)	9 (22%)
Theme3^ǂ^	88 (97%)	2 (2%)	1 (1%)	27 (30%)	38 (42%)	26 (28%)
Total	235 (94%)	11 (4%)	4 (2%)	97 (39%)	89 (36%)	64 (25%)

*Theme 1 = Health needs of marginalized groups

^¶^Theme 2 = Evaluation of projects

^ǂ^Theme 3 = Impact of drug user activism

^₸^Community partners: 1) the BC/Yukon Association of Drug War Survivors, 2) the British Columbia Association for People on Methadone, 3) the Eastside Illicit Drinkers Group for Education, 4) the VANDU’s Tuesday Education Group, 5) SALOME/NAOMI Association of Patients, and 6) the Western Aboriginal Harm Reduction Society

The majority of stakeholders that engaged with VANDU were researchers from local universities and institutes. However, only four studies named community researchers from VANDU or PWUDs as first author. The senior, junior, and other researchers outlined in Table 3 represent a group of authors who, at the time of the publication, held tenure track or other scientific positions affiliated with a university or a lab. VANDU and the community partners are authors who are affiliated with VANDU or other organizations that work under the umbrella of VANDU. Finally, the last category on Table 3, labeled as student/postdoctoral are authors who at the time of publication were identified as students or postdoctoral fellows. The information regarding academic ranking is important because it not only emphasizes the reciprocal knowledge transfer between PWUDs as experts, but it also shows the training of many researchers at the time of publication in the community.

## Discussion

VANDU is one of the first and longest running drug user organizations in North America that has advocated for the health and wellbeing of PWUDs and marginalized members in one of the poorest urban postal codes in Canada. While previous research have shown the effectiveness of programs that are informed, run, or organized by PWUD [19–24], drug user organizations like VANDU seldom been evaluated for their role in research and policy [25]. However, as our review has shown, there is enormous potential for PWUDs and other community researchers to promote advocacy, equity, harm reduction, and inclusion by engaging with similar organizations.

This research has demonstrated that over the past two decades VANDU has facilitated numerous published studies relating to the health of their members and the people from their community. The three themes identified – ‘health needs’, ‘evaluation of projects’, and ‘advocacy’ – are important because they display the scientific output of PWUDs and their community.. The second theme, demonstrates that VANDU members have been at the forefront of harm reduction innovation and mobilization by evaluating and describing their groundbreaking harm reduction programs, such as unsanctioned user-run injection and smoking rooms. Finally, articles that discussed the impact of collective activism and organizing of PWUD indicate the capacity and enormous potential of peers as individuals and groups in health and harm reduction initiatives.

Although not fully evaluated in this research, a closer look at the journal impact factors of the publications that VANDU or its members have been named as co-authors shows the quality and breadth of work that PWUDs have been involved in [26]. For instance, many publications have been featured in top ranking substance use journals such as *Addiction*, *Journal of Urban Health*, *The International Journal of Drug Policy*, *Harm Reduction Journal*, *The Canadian Medical Association Journal*, *AIDS and Behavior*, *The Canadian Journal of Public Health*, and *Social Science & Medicine* to name a few. Moreover, the noted publications showcase the breadth of expertise of VANDU membership, and their innovation to tackle serious public health issues because many of their harm reduction programs, such as unsanctioned peer-run injection room, were mobilized through an organic, member driven processes. The noted unsanctioned user-run injection room was later evaluated through external reviewers via qualitative [27] and cost-effectiveness evaluations [28]. Therefore, VANDU as a user-run advocacy group has shown its enormous depth and innovation, as shown in the research above by implementing interventions that not only improve health and wellbeing of its members, but also through its economic impact by reducing health care costs from overdose and other illness prevented through these programs.

However, we noted that VANDU and its membership have been involved in many projects that did not meet the inclusion criteria of our review – that is, publications did not acknowledge or include them as co-authors. The issue of authorship highlights several barriers that relate to the equity and inclusion of PWUDs. First, the International Committee of Medical Journal Editors [29] defines authorship based on four rigid criteria that would disqualify many PWUDs due to their literacy. However, contributions may be considerable in study design and execution, and interpretation of findings; therefore we recommend changes so that PWUDs’ expertise can be acknowledged through involvement and validity. Second, academic researchers often hold the power to include or not include PWUDs as authors on publications. When considering the power imbalance between the researchers and the participants, these inequalities highlight that participation may not be used to empower PWUDs, but rather to reach the research agendas and advance the careers of academics outside of the community [30–32]. While authorship holds importance for academic researchers in terms of legitimacy and career advancement, it is also important for marginalized communities whose authorship represents the expertise, contributions, and legitimacy of their work. It is also important to emphasize that authorship does not necessarily mean members of the community have been meaningfully engaged. PWUDs may be added to publications to make the work appear community-engaged when in fact their insights have not been integrated and input is tokenistic.

Based on our findings, it is not surprising that PWUDs and others have advocated for greater and more meaningful involvement for members of the DTES. This advocacy is based on legitimizing and valuing PWUDs contributions and expertise, and also to strengthen the inclusion of this community in decisions and actions that affect their lives [33–35].

The “Nothing About Us Without Us” movement has essentially advocated for the broader goals of knowledge transfer where PWUDs have the knowledge and the background to work as researchers “beyond tokenistic involvement in research” [35] p. 6. While previous researchers have laid the foundation for hiring or engaging with PWUD [24, 35] we recommend that future researchers not only acknowledge the participation of PWUDs, but offer capacity building among PWUD to engage them in writing, and authorship – including other avenues of knowledge translation such as media, social media, policy work, and grant writing. We also recommend that every attempt be made to create equitable, fair, and legitimate capacity building opportunities for PWUDs, such as fair access to computers, language, education, and engagement to allow those who show interest to be included. In our own project, it was important to support VANDU by printing out the draft of our manuscript, printing posters, providing the registrations, transportations and financial support so it can be presented orally. As well, it was critical to seek input from its Board and appointed author (HL) at multiple points in time, and to follow up on the outcome of any feedback that was given. We learned that for this group, presenting and using the findings was much more important than the actual writing of the manuscript itself. Along those lines, it is important for peer-reviewed journals to remove some of the rigid requirement for marginalized populations in terms of authorship and access, to not only empower community members, but to reinforce the importance of people with lived experiences as ‘experts’.

Across North America, Europe, Australia, and Asia, PWUDs and other residents of the DTES have been touted as one of the most highly researched populations in the world. In our consultation with VANDU during this process, we noted that several articles that had been published were unknown to VANDU membership. This points to a concern that the results of community-engaged studies are often not disseminated back to this community [36], or leveraged to build the capacity of the community [37].

This study has several limitations that need to be acknowledged. First, some journals did not have standardized reporting for scores related to social networks (e.g., Twitter, Facebook, Reddit, etc.) and article access or downloads. For example, the majority of journals reflected their social media outcomes via Altmetric scores while Elsevier journals reported the outcomes through PlumX metrics which made it extremely difficult to compare. In addition, while BMC, Springer, Sage and Taylor & Francis journals report article downloads/views, this information is not accessible via Wiley and Elsevier journals. Second, the choice of indicators, such as seniority of authors, article access, or Altmetric scores will change with time. For example, many researchers with established rapport with VANDU have moved to more senior academic positions in each subsequent publication. Third, it is important to emphasize some of the shortcomings of the Google Scholar as the only source for identifying articles which may include a lack of information on what constitutes ‘scholarly’ articles, citation overestimations, and unreliable comprehensiveness [38–40]. Fourth, all the articles reviewed show a positive outcome, which could be attributed to publication bias. Moreover, most often VANDU/community members are not versed in quantitative, qualitative and critical analysis that most journals require placing them at significant disadvantage. Also the emphasis on the independence of evaluators in a positivist tradition can obscure or devalue the contributions of community members in evaluation designs, drawing on other approaches to knowledge construction.

Finally, it is important to emphasize that some of the articles highlighted in this review, such as Boyd, & NAOMI Patients Association [33], Demon et al. [36], and Jozaghi & Marsh [41], represent various groups that are supported directly or indirectly through VANDU. For example, 1) the BC/Yukon Association of Drug War Survivors, 2) the British Columbia Association for People on Methadone, 3) the Eastside Illicit Drinkers Group for Education, 4) the VANDU’s Tuesday Education Group, 5) SALOME/NAOMI Association of Patients, and 6) the Western Aboriginal Harm Reduction Society. However, involving the noted groups beyond the VANDU’s board involvement would have been an audience for the work to be presented to, without the stakeholders having the opportunity to get involved in the research. In other words, without the financial support or resources to provide adequate capacity building, asking the above noted groups to contribute as an author in this research would have been counter- productive and would have set the bar for authorship very low. However, we were able to engage HL as the appointed member of VANDU in a meaningful process.

## Conclusions

In summary, this study showcases the range of projects that VANDU has been included in to date, and highlights the capacity, expertise, and knowledge that PWUDs offer to community-engaged work. In the past two decades VANDU has been integral to some of the most pioneering harm reduction projects and research. By fully acknowledging the contributions of PWUDs, research has the power to legitimize the expertise and lived experience that PWUDs bring to various research contexts and ultimately influence policies and other decisions that affect their lives. As other jurisdictions look for ways to tackle health and harm reduction issues related to drug use, including the growing synthetic opioid epidemic [42, 43], it is our hope that activism, advocacy, and community-engaged research can inspire other initiatives to include and acknowledge PWUDs as true partners.

## Abbreviations

DTES: Downtown Eastside
PWUDs: People who use drugs
VANDU: Vancouver Area Network of Drug Users
